# MmpL3 Inhibition as a Promising Approach to Develop Novel Therapies against Tuberculosis: A Spotlight on SQ109, Clinical Studies, and Patents Literature

**DOI:** 10.3390/biomedicines10112793

**Published:** 2022-11-03

**Authors:** Mohd. Imran, Mandeep Kumar Arora, Anurag Chaudhary, Shah Alam Khan, Mehnaz Kamal, Manal Mutlaq Alshammari, Raghad Mohammad Alharbi, Nuha Abdullah Althomali, Ibrahim Mohammed Alzimam, Abdullah Ayed Alshammari, Bashair Hamed Alharbi, Amer Alshengeti, Abdulmonem A. Alsaleh, Shayea A. Alqahtani, Ali A. Rabaan

**Affiliations:** 1 Department of Pharmaceutical Chemistry, Faculty of Pharmacy, Northern Border University, Rafha 91911, Saudi Arabia; 2 School of Pharmaceutical and Population Health Informatics, DIT University, Dehradun 248009, India; 3 Department of Pharmaceutical Technology, Meerut Institute of Engineering and Technology, Meerut 250005, India; 4 Department of Pharmaceutical Chemistry, College of Pharmacy, National University of Science and Technology, Muscat 130, Oman; 5 Department of Pharmaceutical Chemistry, College of Pharmacy, Prince Sattam Bin Abdulaziz University, Al-Kharj 11942, Saudi Arabia; 6 Pharmacy Department, Hotat Bani Tamim General Hospital, Hotat Bani Tamim 16631, Saudi Arabia; 7 Department of Pharmacy, Umm-Alqura University, Makkah 24225, Saudi Arabia; 8 Department of Community Pharmacy, Kol Alosra, Taif 26512, Saudi Arabia; 9 Faculty of Pharmacy, Northern Border University, Rafha 91911, Saudi Arabia; 10 Nahdi Medical Company, Riyadh 12211, Saudi Arabia; 11 Department of Clinical Pharmacy, Umm-Alqura University, Jeddah 21921, Saudi Arabia; 12 Department of Pediatrics, College of Medicine, Taibah University, Al-Madinah 41491, Saudi Arabia; 13 Department of Infection Prevention and Control, Prince Mohammad Bin Abdulaziz Hospital, National Guard Health Affairs, Al-Madinah 41491, Saudi Arabia; 14 Clinical Laboratory Science Department, Mohammed Al-Mana College for Medical Sciences, Dammam 34222, Saudi Arabia; 15 Medical Laboratory Department, Erhadh Hospital, Dammam 32434, Saudi Arabia; 16 Molecular Diagnostic Laboratory, Johns Hopkins Aramco Healthcare, Dhahran 31311, Saudi Arabia; 17 College of Medicine, Alfaisal University, Riyadh 11533, Saudi Arabia; 18 Department of Public Health and Nutrition, The University of Haripur, Haripur 22610, Pakistan

**Keywords:** tuberculosis, drug-resistance, Mmpl3, SQ109, clinical studies, patent

## Abstract

Tuberculosis (TB) is accountable for considerable global morbidity and mortality. Effective TB therapy with multiple drugs completes in about six months. The longer duration of TB therapy challenges patient compliance and contributes to treatment collapse and drug resistance (DR) progress. Therefore, new medications with an innovative mechanism of action are desperately required to shorten the TB therapy’s duration and effective TB control. The mycobacterial membrane protein Large 3 (MmpL3) is a novel, mycobacteria-conserved and recognized promiscuous drug target used in the development of better treatments for multi-drug resistance TB (MDR-TB) and extensively drug-resistant TB (XDR-TB). This article spotlights MmpL3, the clinical studies of its inhibitor (SQ109), and the patent literature. The literature on MmpL3 inhibitors was searched on PubMed and freely available patent databases (Espacenet, USPTO, and PatentScope). SQ109, an analog of ethambutol (EMB), is an established MmpL3 inhibitor and has completed Phase 2b-3 clinical trials. Infectex and Sequella are developing orally active SQ109 in partnership to treat MDR pulmonary TB. SQ109 has demonstrated activity against drug-sensitive (DS) and drug-resistant (DR) *Mycobacterium tuberculosis* (*Mtb*) and a synergistic effect with isoniazid (INH), rifampicin (RIF), clofazimine (CFZ), and bedaquiline (BNQ). The combination of SQ109, clofazimine, bedaquiline, and pyrazinamide (PZA) has been patented due to its excellent anti-TB activity against MDR-TB, XDR-TB, and latent-TB. The combinations of SQ109 with other anti-TB drugs (chloroquine, hydroxychloroquine, and sutezolid) have also been claimed in the patent literature. SQ109 is more potent than EMB and could substitute EMB in the intensive stage of TB treatment with the three- or four-drug combination. Developing MmpL3 inhibitors is a promising approach to fighting the challenges associated with DS-TB and DR-TB. The authors foresee MmpL3 inhibitors such as SQ109 as future drugs for TB treatment.

## 1. Introduction

Tuberculosis (TB) is a contagious bacterial illness known to humankind since ancient times. The causal microorganism of TB is *Mycobacterium tuberculosis* (*Mtb*). Lung or pulmonary TB is the most common form, but *Mtb* also affects other body parts [1]. TB does not spare any age group and is omnipresent worldwide [1]. Most TB patients remain asymptomatic (latent TB) and non-contagious. However, approximately 10% of latent TB cases may advance to active TB (active or symptomatic TB) [2]. Some usual symptoms of active TB comprise continuing chronic cough, hemoptysis, night sweating, and weight loss [1,2,3]. Active TB is associated with a high mortality rate if left untreated [1,2,3]. The 2021 TB report of the World Health Organization (WHO) states that TB is one of the top 10 reasons for global deaths, about one-quarter of the global population is affected by *Mtb*, and the global burden of TB is expected to increase due to the COVID-19 pandemic [4].

Pulmonary active TB, unlike latent TB, is highly contagious and spreads through the air by the coughing, spitting, speaking, or sneezing of active TB patients. Extrapulmonary TB infections, such as skeletal TB, brain (meningitis) TB, joint TB, gastrointestinal TB, liver TB, genitourinary TB, cutaneous TB, etc., show a wide range of symptoms [5,6]. Heavy smokers, and HIV/AIDS and other immune-compromised patients are more prone to acquire an active infection [5]. Active TB infections are diagnosed using chest x-rays and the microscopic examination of the body fluid. In contrast, blood and tuberculin skin tests (TST) are employed to diagnose latent TB [6]. BCG vaccination, screening of populations at high risk, and early diagnosis and treatment are some ways to prevent and combat TB [7]. Conventional anti-TB drugs are classified into first-line, e.g., isoniazid (INH), rifampicin (RIF), ethambutol (EMB), pyrazinamide (PZA) and second-line regimens such as aminoglycosides (streptomycin), fluoroquinolones (moxifloxacin), clofazimine (CFZ), linezolid, ethionamide, and prothionamide [4,8,9,10]. Anti-TB drugs are frequently used in combination (multiple drugs) over a long period (6–9 months), but the duration of therapy varies according to the type of TB [4,8,9,10]. Irrational use of anti-TB drugs has resulted in developing resistance to anti-TB drugs. In multi-drug resistant TB (MDR-TB), resistance develops to the two most commonly used first-line drugs (INH and RIF). In extensively drug-resistant TB (XDR-TB), in addition to RIF and INH, resistance develops to two or more antibiotics belonging to fluoroquinolones and aminoglycosides [4,10,11]. Recently total drug-resistant TB (TDR-TB) has also emerged [12].

The longer therapy with anti-TB medications leads to unavoidable adverse effects. For example, the first-line drugs (INH, RIF, EMB, and PZA), bedaquiline and pretomanid can cause hepatotoxicity [12,13]. The longer duration of TB therapy challenges patient compliance and contributes to therapy failure and the progress of drug-resistant (MDR, XDR, and TDR) strains of *Mtb*. These strains are particularly dangerous for immunocompromised people. The irrational use of anti-TB drugs also promotes the development of MDR-TB, XDR-TB, and TDR-TB [4,10,11,12,13]. Therefore, new treatments with an inventive mechanism of action are desperately desired to shorten the TB therapy’s duration and effective TB control. Accordingly, scientists have identified novel drug targets (DprE1, QcrB, MmpL3, etc.) for anti-TB drug development [12,13]. The mycobacterial membrane protein Large 3 (MmpL3) is a novel, mycobacteria-conserved, and promiscuous drug target developed to provide better therapy for TB. This article spotlights MmpL3, the clinical studies of its inhibitor, and the patent literature.

## 2. Mycobacterial Membrane Protein Large 3 (MmpL3)

**Figure 1:**
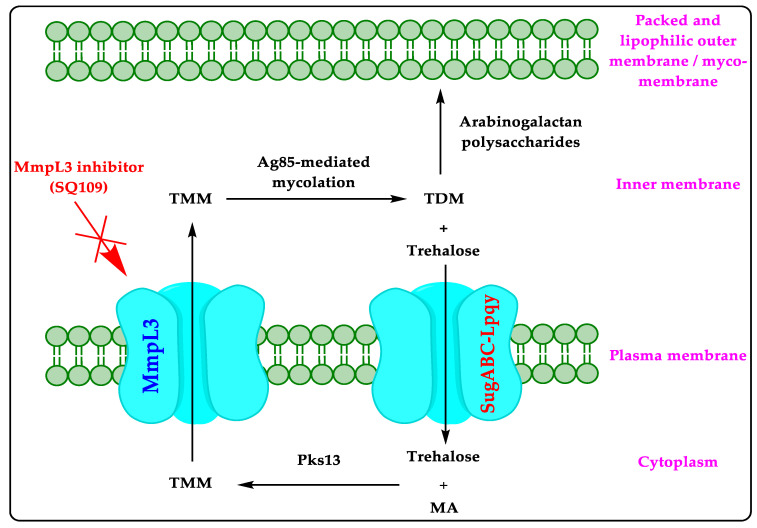
Mechanism of action of MmpL3 inhibitor (SQ109).

MmpL is a transport protein (trehalose monomycolate flippase) in *Mtb*. There are about 13 MmpLs, but only MmpL3 is essential in *Mtb* [14]. The physiological role, structure, and properties of MmpL3 are well described in the literature [14,15,16,17,18,19]. Mycolic acid (MA) is an essential component of the MA-based hydrophobic outer cell wall of *Mtb* [14,15,16,17,18,19]. MA is produced in the cytoplasm of the *Mtb* cell. In the cytoplasm of the *Mtb*, the type I polyketide synthase 13 (Pks13) drives the interaction of MA and trehalose to produce trehalose monomycolate (TMM). MmpL3 is located at the plasma membrane of the *Mtb*. MmpL3 is responsible for transporting TMM from the cytoplasm to the inner membrane of the *Mtb*. In the inner membrane, Ag85 mediates the conversion of TMM to trehalose dimycolate (TDM) and trehalose. The trehalose returns to the cytoplasm via the SugABC-Lpqy transporter to restart this cycle. The TDM makes the covalent bond with the arabinogalactan polysaccharides to synthesize a packed and MA-based hydrophobic outer cell wall of *Mtb* (Figure 1).

The MA-based hydrophobic outer cell wall of *Mtb* is impermeable to many chemical compounds, including antibiotics, and protects and contributes to the pathogenic success of *Mtb* [14,16]. Therefore, the expression of MmpL3 is essential for *Mtb*’s survival and pathogenicity [14,15,16,17,18,19]. The inhibitors of MmpL3 (SQ109) cause the diminution of MmpL3, which leads to the cessation of cell wall synthesis, cell division, and thevrapid death of *Mtb* [14,15,16,17,18,19].

## 3. Literature on MmpL3 Inhibitors

The literature on MmpL3 inhibitors was searched on the PubMed database using “MmpL3” as a keyword on 1 October 2022. This search provided 146 articles, including 21 review articles. The summary of 12 relevant and recent review articles is mentioned in Table 1. The authors did not find an MmpL3 inhibitor-based review article discussing clinical studies on MmpL3 inhibitors (SQ109), the development of SQ109, or the patent literature of MmpL3. This aspect provides novelty to the current review article over the previously published reviews on MmpL3 inhibitors.

## 4. Clinical Studies on MmpL3 Inhibitors

Studies [14,16,19,20,23,24,25,26,27,28,29] have disclosed the chemistry of some important inhibitors of MmpL3 (SQ109, NITD-304, NITD-349, AU1235, CRS400393, BM212, THPP, spiropiperidine, TBL-140, ICA38, HC2091, BM533, BM635, rimonabant, C215, and PIPD1). The clinical studies on MmpL3 inhibitors were searched on 1 October 2022, on the clinicaltrial.gov database [30] utilizing different keywords (SQ109 = 7 studies; No anti-TB study found for NITD-304, NITD-349, AU1235, CRS400393, BM212, THPP, spiropiperidine, TBL-140, ICA38, HC2091, BM533, BM635, rimonabant, C215, or PIPD1). A general search on PubMed was also conducted with the earlier mentioned keywords in the clinical trial and randomized controlled trial section of PubMed. Three studies were identified for the “SQ109” keyword [31,32,33], while other keywords did not produce clinical studies related to TB. The summary of clinical studies on SQ109 is provided in the SQ109 section of this study.

## 5. SQ109

**Figure 2:**
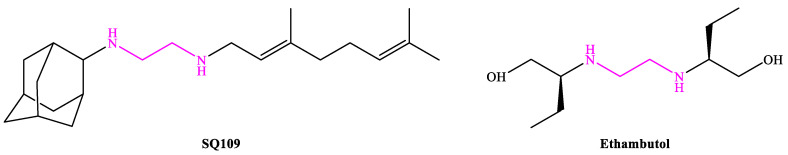
Chemical structures of SQ109 and ethambutol.

SQ109 (Synonym: NSC722041; Molecular Formula: C_22_H_38_N_2_; Molecular Weight: 330.55; CAS registry number: 502487-67-4; ChemSpider ID: 4438718; PubChem substance ID: 175426955; PubChem CID: 5274428; Figure 2) is a 1,2-ethylenediamine-based analog of EMB (Figure 2) [34,35]. The 1,2-ethylenediamine linker is essential for the anti-TB activity of SQ109 and EMB [36].

**Figure 3:**
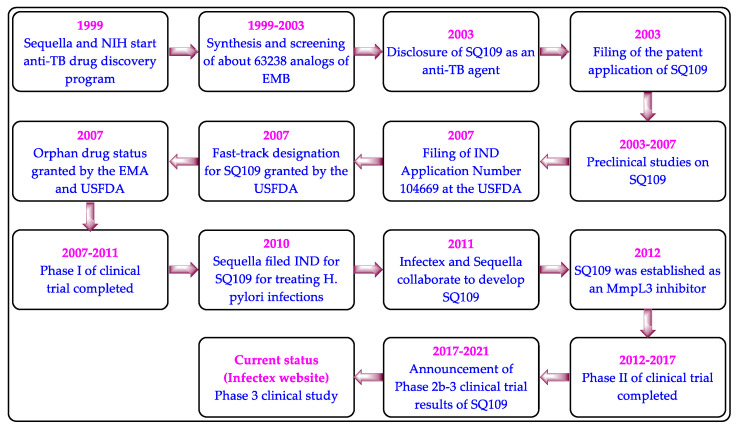
The development timeline of SQ109.

Infectex and Sequella are developing SQ109 in partnership to treat MDR pulmonary TB [37,38]. SQ109 was developed by focusing on EMB, but they share an uncommon chemical skeleton (Figure 2) and mechanism of action. The chemical structure, synthesis, anti-TB activity, oral bioavailability, and acid stability of SQ109 were first disclosed in 2003 [34,39]. The development timeline of SQ109 is presented in Figure 3.

### 5.1. Mechanism of Action

SQ109 demonstrates its anti-TB activity through three different mechanisms comprising inhibition of MmpL3 (Figure 1), biosynthesis of quinones (MenG and MenA), and a reduction in ATP synthesis in *Mtb* [37,38,39,40].

### 5.2. Preclinical Studies

The preclinical studies have established the efficacy of SQ109 against all forms of TB, including DS-TB, DR-TB, and the slow vegetative form of *Mtb* (latent-TB) [38,39] (Table 2). SQ109 has also shown activity against *Aspergillus fumigatus*, *Candida* spp., *Helicobacter pylori*, *Haemophilus influenzae*, and *Streptococcus pneumoniae* [38]. SQ109 presented low oral bioavailability but achieved 45-fold higher concentrations in TB murine target organs (lung and spleen) than in plasma, reduced TB-treatment time by about 25–30% in in vivo models, and exhibited synergistic effects with INH, RIF, CFZ, and BDQ [37,38,39] (Table 2).

### 5.3. Clinical Studies on SQ109

The clinical studies on SQ109 were searched on the clinicaltrial.gov database [30] and PubMed. The summary of clinical studies on SQ109 is provided in Table 3.

Among the seven clinical studies on SQ109 (Table 3), the results of NCT01218217 [31] and NCT01785186 [32] have been published. The NCT01218217 study was conducted between December 2010 and August 2011. It was observed that SQ109 (75, 150, and 300 mg) alone or in combination with RIF was safe and tolerable. The NCT01785186 was conducted between May 2013 and March 2014. However, the recruitment for the SQ109 group was halted because of the unmet pre-specified efficacy threshold. Accordingly, this study is silent about the data of the SQ109 monotherapy. However, the 2b study advocated enhanced activity of SQ109 when added to the MDR regimen [22]. In another published study [33], TB patients were treated with SQ109, RIF, and a combination of RIF and SQ109 for 14 days. The sputum of the treated patients was collected daily and analyzed for the colony-forming units (CFU). It was observed that RIF significantly reduced the CFU more than SQ109. The adverse effects associated with SQ109 are mentioned in one clinical study [31] and the literature [37,38,39]. The common side effects of SQ109 included dose-dependent mild gastrointestinal discomfort, nausea, and vomiting, which did not require any clinical intervention. These studies did not reveal any relevant impact on QT prolongation, neurological parameters, vital signs, blood chemistry, or ophthalmological data [31,37,38,39].

## 6. Patent Searching and Analysis

The patent searching was done on 1 October 2022, by the freely available patent databases (Espacenet, United States Patent and Trademark Office (USPTO), and PatentScope) [41,42,43,44] utilizing different keywords (SQ109 and MmpL3). The patent literature search provided many hits on the Espacenet database (SQ109: 218 hits; MmpL3: 119 hits), USPTO database (SQ109: 115 hits; MmpL3: 39 hits), and PatentScope database (SQ109: 199 hits; MmpL3: 140 hits). The duplicate patents/patent applications were removed and segregated based on their patent family. The patents/applications explicitly stating the application of MmpL3 inhibitors or SQ109 to prevent/treat TB were identified and analyzed.

**WO2003096989A2** (Assignee: National Institute of Health) disclosed 1,2-ethylenediamine-based analogs of EMB to provide new molecules for drug-resistant TB and shorten the treatment of TB [45]. **WO2003096989A2** is the first patent application disclosing SQ109 and SQ109 dihydrochloride [45]. The compositions of SQ109 to treat *Mtb* infections are also specifically patented in **US8268894B2** [46] and **US7842729B2** [47], which are family members of **WO2003096989A2** [45].

**US7456222B2** (Assignee: Sequella; Status: Patented case) claims a method of treating TB utilizing a therapeutically effective amount of SQ109 (1 to 1000 mg), optionally with a pharmaceutically acceptable carrier [48]. This patent disclosed the synthesis, structure elucidation data (NMR and mass spectrum), the anti-TB activity of SQ109 in a mice model of TB, the efficacy of combinations of SQ109 with other anti-TB drugs (INH and RIF), and MIC values of SQ109 for different bacteria (Gram-Positive and Gram-Negative), anaerobes, fungi, and *Mtb*. This patent states that the dihydrochloride salt of SQ109 was used in most of the experiments; the maximum tolerated dose of SQ109 is 600 mg/kg, whereas an 800 mg/kg dose is fatal; SQ109 has low oral bioavailability (3.8%); SQ109 primarily distributes into the lungs and spleen; plasma protein binding of SQ109 is concentration-dependent (non-linear) due to saturation of the protein binding sites and fluctuates from 15% (20 ng/mL) to 74% (200 ng/mL) to 48% (2000 ng/mL); a low concentration of SQ109 (1–10 mg/kg) shows activity equal to that of EMB at 100 mg/kg; the combination of SQ109 with INH or RIF showed synergistic effects over INH or RIF alone; SQ-109 is equally active against DS and DR strains of *Mtb*, and SQ109 could substitute EMB in the serious stage of TB treatment comprising combination therapy. It further states that SQ-109 revealed decent activity against *Candida albicans* (MIC = 4–8 ug/mL) [48]. **US8202910B2** [49] is a family member of **US7456222B2** [48] with an analogous specification. **US8202910B2** claims a composition comprising SQ109 and other therapeutic agents like INH, RIF, and antiparasitic, and antiviral drugs. It also relates to treating fungal diseases using SQ109 or its combinations [49].

**US10576079B2** (Assignee: University of California; Status: Patented case) relates to a pharmaceutical composition for treating TB (MD-TB, XDR-TB, and latent-TB) using a combination of CFZ, BDQ, PZA, and SQ109. The in vitro intramacrophage *Mtb*-iGFP model showed a 98.2% inhibition for the combination of CFZ (0.104 μg/mL), BDQ (0.0085 μg/mL), PZA (15 μg/mL), and SQ109 (0.25 μg/mL) [50].

The patent search also exposed some patents/patent applications claiming SQ109 inventions. However, these documents provide little information about the experimental facts of the claimed SQ109 inventions (Table 4).

**Table 4:** Patents/patent applications of SQ109 without experimental details of the claimed SQ109 inventions.

S. No.	Patent/Patent Application Number (Assignee; Status)	Summary of the Claimed Invention
1	**WO2021090283A1** (Foundation for Neglected Disease Research; No national phase entry)	A method of reducing the duration of TB treatment in patients suffering from DS-TB or DR-TB utilizing a combination of chloroquine/hydroxychloroquine and an anti-TB agent (SQ109, BNQ, macozinone, CFZ, etc.). The use of the claimed combination to reduce the relapse of TB in patients co-infected with HIV-1 is also claimed [51].
2	**WO2018191628A1** (University of California; No national phase entry)	An anti-TB pharmaceutical composition of CFZ, BZQ, and PZA that may optionally contain a therapeutically effective amount of SQ109 [52].
3	**WO2017191444A1** (UCL Business PLC; No national phase entry)	The polymersome (vesicles formed from amphiphilic block copolymers) composition of anti-TB drugs (INH, RIF, SQ109, etc.) with improved in vivo anti-TB activity over the free drug [53].
4	**US20170189474A1** (Cedars-Sinai Medical Center; Abandoned)	A method of treating Hirschsprung-associated fungal enterocolitis with an antifungal agent (SQ109, posaconazole, itraconazole, etc.) [54].
5	**US2017136102A1** (GangaGen Inc.; Abandoned)	A method of treating *Mtb* infection with a combination of an outer membrane acting biologic and an anti-TB drug (SQ109, INH, RIF, EMB, PZA, etc.), wherein the outer membrane acting biologic increases the permeability of the mycobacteria outer membrane for the anti-TB drug [55].
6	**EP2340022B1** (Pfizer; Invalid due to non-payment of fee)	A combination of sutezolid with at least two anti-TB drugs selected from the groups consisting of INH, RIF, PZA, CFZ, SQ109 etc., to treat TB [56].
7	**US2021069156A1** (Cornell University; Non-final action mailed)	A method of treating inflammatory disease (inflammatory bowel disease) in a subject diagnosed with one or more loss-or-function mutations in the CX3CR1 gene using a therapeutically effective amount of an anti-fungal agent (SQ109, voriconazole, fluconazole, etc.) [57].
8	**US10624893B2** (GlaxoSmithKline; Patented case)	A method for the treatment of TB using a therapeutically effective amount of GSK2556286 optionally in combination with other drugs (SQ109, INH, RIF, and PZA) [58].
9	**US9572809B2** (Spero Trinem; Patented case)	A method of controlling, treating, or reducing the advancement, severity or effects of a mycobacterium disease (TB) using a combination of VXc-486 and another antibiotic (SQ109, CFZ, sutezolid, PZA, and INH) [59].

Our search also revealed patents/patent applications claiming MmpL3 inhibitors of diverse chemical classes, including indole derivatives [60,61,62,63,64,65,66,67,68], indazoles [69], adamantane derivatives [70,71,72], ethylene diamines [73], trehalose analogs [74], and benzothiazole amide [75] for preventing/treating TB. The compounds claimed in these patents/applications have not been clinically tested. Therefore, the authors have not summarized them. However, these patent documents would be worthy of review for those interested in discovering MmpL3 inhibitors.

## 7. Discussion

Despite innovative development in the diagnosis and healthcare system, the emerging cases of MDR-TB (long treatment of up to two years) and XRD-TB (long and costly treatment due to limited treatment options) are posing questions about the current TB-therapy regimen and making TB an unmet medical need [14,16,22]. Moreover the COVID-19 pandemic has reversed the progress of global TB-control programs [4,16]. These concerns warrant developing potent anti-TB treatments with an inventive mechanism of action, high chemical stability, lower toxicity profile, and shorter treatment duration [14,24]. Accordingly, scientists have identified and validated novel drug targets (MmpL3, DprE1, QcrB, etc.) for anti-TB drug development [12,13].

MmpL3 inhibition weakens *Mtb*’s cell wall and steers cell death [14,16,20,21,60]. MmpL3 is highly conserved in mycobacteria as well as in corynebacterial but is absent in humans. This motivating feature of MmpL3 suggests the absence of a mechanism of action-based side effects in humans [14,51]. The MmpL3 inhibitors are also active against non-tubercular mycobacteria (NTM), including *M. abscessus* and *M. leprae* [14,23,61]. The treatment options for NTM infections are limited and poor, with higher chances of adverse effects and relapse [23]. This also encourages the development of MmpL3 inhibitors. The inhibitors of MmpL3 have demonstrated synergistic effects with many anti-TB drugs (INH, RIF, CFZ, and BDQ) [21,23]. The synergist effect permits the use of lower doses of drugs and reduces the possibility of drug resistance. This attribute also makes MmpL3 an attractive therapeutic target against *Mtb* infections. The MmpL3 inhibitors do not disclose a common pharmacophore. Many chemical classes (ethylenediamine derivatives, carboxamide derivatives, benzothiazole amides, adamantyl ureas, pyrroles and pyrazoles, benzimidazoles, spiropiperidines, and piperidinol) of MmpL3 are reported [14,16,19,20,23,24,25,26,27,28,29]. Consequently, MmpL3 is recognized as a promiscuous drug target [16,21]. Being a promiscuous target, repurposing studies on USFDA-approved molecules are foreseeable to identify MmpL3 inhibitors. The drug-repurposing studies will also help identify non-toxic nutraceuticals that can be administered with current regimens of TB therapy [14]. The MmpL3 belongs to the resistance, nodulation, and cell division (RND) transporter family, which depends on proton motive force (PMF) to transfer substrates from the cytoplasm to the inner membrane of the *Mtb* [24,68]. Accordingly, the activity of MmpL3 is driven by the electron transport chain (ETC) and the PMF in the *Mtb* [16]. The additional PMF-disrupting effects of some MmpL3 inhibitors (SQ109) potentiate their anti-TB activity against replication and non-replicating *Mtb* [23]. Therefore, the authors trust that combining QcrB inhibitors and MmpL3 inhibitors may provide highly synergistic effects. The development of MmpL3 inhibitors faces some challenges. Only a few in vitro and whole cell-based assays allowing the recognition of MmpL3 inhibitors are available. The absence of simple and relatively high-throughput assays to quickly screen potential MmpL3 inhibitors currently denotes a hindrance to further development [60]. The conformational changes or alterations in the transporter structure of MmpL3 are possibly causing mutation in MmpL3, which may cause drug resistance [16,63,68]. This aspect needs to be considered while developing MmpL3 inhibitors. Chemically, the most important MmpL3 inhibitors contain lipophilic groups (cycloalkyl, adamantyl, etc.). This aspect improves penetration into the *Mtb*’s cell wall [24]. However, this property may also cause problems of low aqueous solubility, increased protein binding, and toxicity. These issues may hinder the development of suitable dosage forms of these MmpL3 inhibitors. Long-term TB therapy is needed to avoid relapse of TB due to genetically DR bacteria or phenotypic drug tolerance (PDT). The PDT develops due to the induction of efflux pumps in *Mtb*. Accordingly, studying phenotypic drug tolerance concerning MmpL3 inhibitors is also warranted to develop better MmpL3 inhibitors [51].

SQ109 (a 1,2-ethylene diamine compound) is the most advanced and promising oral MmpL3 inhibitor under clinical development (phase II-3b trial completed). SQ109 possesses an appreciable tolerability, safety, and efficacy profile against all forms of TB, including pulmonary TB, MDR-TB, XDR-TB, genitourinary-TB, nervous system TB, osseous-articular system TB, and TB of the lymph glands [14,60]. Based on the clinical studies data (Table 3), the authors believe that tablet formulation of dihydrochloride salt of SQ109 may be the marketed dosage in the future. A low dose of SQ109 demonstrated remarkable synergy with first-line drugs (INH and RIF) and bedaquiline. These combinations also shortened the treatment duration by 25–30% [37]. The replacement of EMB with SQ109 in TB therapy has also exhibited improved efficacy and shorter treatment duration. This characteristic makes SQ109 a unique drug to become an excellent component of the combination of TB therapy [37]. SQ109 is also reported to interfere with the ETC (menaquinone inhibitor) and ATP synthesis in *Mtb*. These additional mechanisms make SQ109 an excellent anti-TB drug [14]. We believe combining QcrB inhibitors like telacebec with SQ109 may be synergistic and advantageous. A recent study has disclosed six metabolites of SQ109, including a hydroxy adamantyl analog of SQ109 and N′-adamantylethylenediamine. None of these metabolites showed anti-TB activity against *Mtb*, suggesting that the metabolites of SQ109 do not contribute to the anti-TB activity of SQ109 [76]. The spontaneous resistance to SQ109 has not yet been reported in *Mtb*. However, modest cross-resistance to SQ109 species containing MmpL3 has been reported [71]. The bacterial mutation rate for SQ109 is quite low. Therefore, the chances of *Mtb*’s resistance development to SQ109 are thin [37,38]. SQ109 has also demonstrated appreciable activity against *H. pylori* and *C. albicans*. SQ109 exhibited the killing of 99.99% of *H. pylori* with concentrations easily achievable in stomach contents and tissues [37,38]. These microorganisms do not have MmpL3 genes. This suggests the activity of SQ109 against *H. pylori* and *C. albicans* involves a different mechanism [71]. These findings suggest the incorporation of SQ109 in *H. pylori* combination therapy and using SQ109 as an antifungal agent.

**Figure 4:**
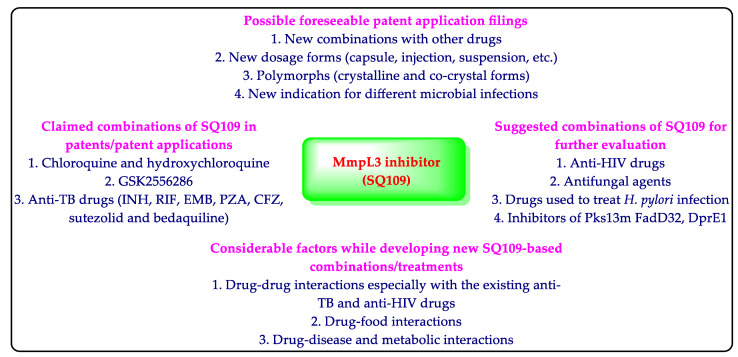
Summary and suggestions for the SQ109 -based work.

A company files patent applications to protect and get the commercial benefits of its inventions [42,43,77]. Sequella has also protected SQ109-based inventions related to the pharmaceutical compositions/combinations and methods of treating *Mtb* infections utilizing SQ109 [45,46,47,48] (Figure 4). Sequella’s patents/patent applications provide experimental details of the claimed SQ109 inventions. However, many patents/patent applications do not provide experimental details of the claimed compositions of SQ109 with other drugs [51,54,56,57,58]. Therefore, the authors recommend carrying out the experiments using the claimed combinations of SQ109 with other drugs provided in such patent applications [51,54,56,57,58] (Figure 4). This will help to identify more SQ109-based anti-TB combinations. The HIV-patients are prone to developing TB. SQ109 has demonstrated good activity against *H. pylori* and fungi. Therefore, the combinations comprising SQ109 and other drugs (anti-HIV and antifungal) may also be tested (Figure 4). The study of drug interaction helps to avoid pharmacokinetic and pharmacodynamic-related issues of a drug [42,43]. Accordingly, an assessment of the SQ109-based drug interaction studies is also necessary (Figure 4). A company can file different types of patents for a drug [12,77]. Therefore, the writers foresee the advance and patent filings of further SQ109-based inventions comprising new combinations of SQ109 with other drugs (anti-HIV, antifungal, and different antibiotics/antivirals), new dosage forms of SQ109 (capsule, injection, suspension, etc.), polymorphs of SQ109 (new crystalline or co-crystal forms), and new indications of SQ109.

## 8. Conclusions

MmpL3 is a validated and mycobacteria-conserved promiscuous drug target in the development of drugs against MDR-TB, XDR-TB, and NTM. SQ109 is the most advanced orally active EMB-based MmpL3 inhibitor in clinical trials. SQ109 is more potent than EMB and demonstrated synergistic effects with first-line anti-TB drugs (INH and RIF), in addtion to a shorter treatment duration. SQ109 is a potential candidate to replace EMB from the currently approved anti-TB regimens. The development of SQ109-based treatments for *H. pylori* and fungal infections is also predictable. The patent literature discloses some good inventions of SQ109 with some antimicrobial agents. The authors trust the potential of the MmpL3 inhibitor (SQ109) to improve anti-TB drug regimens and foresee tremendous scope for developing SQ109-based antimicrobial regimens.

## Figures and Tables

**Table 1:** Summary of relevant and recent review articles on MmpL3.

Ref. No.	Year	Summary of the Review Article
[14]	2020	Reviews MmpL3 (physiological role, structure, and properties) and ligands/inhibitors of MmpL3 (AU1235, ICA38, SQ109, rimonabant, SPIRO, and NITD-349).
[16]	2021	Describes MmpL3 as a drug target, different chemical classes of MmpL3 inhibitors (derivatives of indole carboxamide, pyrrole/pyrazole, quinoline/quinolone, adamantane, benzimidazole, acetamide, and spiro-compound) and different ligands of MmpL3 like ICA38 (PDB ID: 6AJJ), rimonabant (PDB ID: 6AJI), SQ109 (PDB ID: 6AJG), and U1235 (PDB ID: 6AJH).
[20]	2022	Reviews indole derivatives as MmpL3 inhibitors (NITD-349, indolamide, adamantanol analogs, and indole-2-carboxamides) and inhibitors of other anti-TB drug targets (InhA, DprE1, KasA, chorismate mutase, DNA replication, DNA gyrase, dihydrofolate reductase).
[21]	2022	Describes MmpL3 as a promiscuous drug target and also spotlights the MmpL3 inhibitors of different chemical classes (adamantyl derivatives, piperidinol derivatives, and benzimidazole derivatives). It also highlights other anti-TB drug targets (TrmD, Ag85C, GyrB, and ClpC1).
[22]	2021	Briefly explains clinical study data (NCT01785186) of SQ109 (an MmpL3 inhibitor) and comments on the improved anti-TB activity of SQ109 with MDR regimens.
[23]	2020	Talks about MmpL3, the preclinical/clinical development of MmpL3 inhibitors (BM212, THPP, SQ109, Spiro, NITD-349, NITD-304, AU1235, C215, and HC2091), and different chemical classes of MmpL3 inhibitors (derivatives of indole, benzimidazole, benzothiazole, piperidine, 4-Thiophen-2-yloxane-4-carboxamide, benzofuran, quinoline/quinolone, naphthalene, acetamide, and pyrrole).
[24]	2020	Explores the current development of MmpL3 inhibitors (ethylenediamine derivatives, carboxamide derivatives, benzothiazole amides, adamantyl ureas, pyrroles and pyrazoles, benzimidazoles, spiropiperidines, and piperidinol), along with their structure-activity relationship (SAR) and challenges in developing them. It also provides the chemical structure of many MmpL3 inhibitors (AU1235, CRS400393, BM212, THPP, spiropiperidine, TBL-140, ICA38, HC2091, BM533, BM635, rimonabant, C215, PIPD1, NITD-349, NITD-304, and SQ109,).
[25]	2020	Surveys the new targets for TB, including MmpL3 and the chemistry of MmpL3 inhibitors (design and structural features) in clinical/preclinical trials.
[26]	2020	Identified lead compounds from PubChem database targeting MmpL3 and other anti-TB drug targets by high-throughput screening.
[27]	2019	Underlines the chemical structures and designs of MmpL3 inhibitors.
[28]	2018	Highlights the target validation, discovery, hit-optimization, and SAR of MmpL3 inhibitors of different chemical classes (ethylenediamine, adamantyl ureas, phenyl pyrroles, benzimidazoles, indole carboxamides, and spiropiperidines).
[29]	2014	Discloses MmpL3 as a validated target for developing anti-TB medications. It also discloses SQ109 and BM212 as MmpL3 inhibitors.

**Table 2:** The anti-TB activity of SQ109 and its combinations against *Mtb*.

The Anti-TB Activity of SQ109			The Anti-TB Activity of SQ109 Combinations in Mice		
Susceptibility Profile	Assay	MIC (μg/mL)	Drug Regimen	Log_10_ CFU in Lung	Log Decrease
H37Rv (pan-susceptible)	BACTEC	≤0.2	**Two weeks**		
H37Rv (pan-susceptible)	Alamar	≤0.39	Untreated	6.16 ± 0.02	-
Erdman (pan-susceptible)	Alamar	≤0.39	INH + RIF + EMB	4.64 ± 0.23	1.52
EMB-resistant	Alamar	0.78	INH + RIF + SQ109	4.46 ± 0.12	1.70
INH-resistant	Alamar	0.78	**Four weeks**		
RIF-resistant	Alamar	≤0.39	Untreated	6.42 ± 0.76	-
XDR plus EMB-resistant	Microbroth	0.20	INH + RIF + EMB	3.86 ± 0.14	2.56
			INH + RIF + SQ109	3.26 ± 0.12	3.16

**Table 3:** Summary of clinical studies on SQ109.

Title (Allocation; Intervention Model; Masking; Purpose)	Intervention and Active Comparator (AC)	NCT Number (Status; Phase; Number Enrolled; Results; Outcome Measures)	Sponsor/Collaborator (Location; Study Start Date (SSD); Study Completion Date (SCD); Last Update Date (LUD))
Pharmacokinetics and early bactericidal activity (EBA) of SQ109 in adult subjects with pulmonary TB (Randomized; Parallel assignment; None (Open-label); Treatment of TB)	SQ109 monotherapy (75 mg, 150 mg, and 300 mg tablet daily) or a combination of RIF with SQ109 (RIF standard dose + 150 mg or 300 mg of SQ109) for 14 days; AC: RIF capsule (150 mg)	**NCT01218217** (Completed; 2; 90; Not available; EBA of SQ109 monotherapy and combination therapy of SQ109 with RIF)	Michael Hoelscher and Sequella, Inc. (South Africa; November 2010: May 2012; 14 January 2013)
Evaluation of SQ109 plus PPI in urea breath test-positive volunteers (Not mentioned; Single group assignment; None (Open-label); Treatment of *H. pylori* infection)	SQ109 (300 mg) daily for two weeks; AC: Not mentioned	**NCT01252108** (Withdrawn due to lack of funding: 2: 0: Not available; safety and efficacy of SQ109 against *H. pylori* infection in adult patients)	Sequella, Inc. (Not mentioned; March 2012; August 2015; 17 November 2015)
Evaluation of SQ109, high-dose RIF, and moxifloxacin in adults with smear-positive pulmonary TB in a MAMS design (Randomized: Single group assignment: None (Open-label): Treatment of TB)	Combinations of SQ109 (300 mg) with RIF (10 to 35 mg/kg), INH (75 mg), PZA (400 mg) and pyridoxine (25 mg); AC: Combination of INH, RIF, PZA, and EMB	**NCT01785186** (Completed: 2: 365; Available; Two negative sputum cultures utilizing liquid media)	Michael Hoelscher and Sequella, Inc. (South Africa; April 2013; March 2015; 20 September 2017)
Escalating single-dose safety, tolerability, and pharmacokinetics of SQ109 in healthy volunteers (Randomized; Single group assignment; Quadruple (participant, care provider, investigator, outcomes assessor); Treatment of TB)	A single oral dose of SQ109 (10 mg, 20 mg, 50 mg, 100 mg, 200 mg, 300 mg, and the combination of fatty food with 300 mg of SQ109); AC: Placebo	**NCT01585636** (Completed; 1; 62; Not available; Safety and pharmacokinetics of single dose of SQ109 for seven days)	Sequella, Inc. and Quintiles, Inc. (United States; September 2006; February 2007; 19 August 2013)
Dose escalation study of SQ109 in healthy adult volunteers (Randomized; Parallel assignment; Double (participant, investigator); Treatment of MDR-TB)	SQ109 (75 mg and 150 mg) daily for 14 days and SQ109 (150 mg) daily on days 1–5, 9, and 14; AC: Placebo	**NCT00866190** (Completed; 1; 10; Not available; Safety and tolerability evaluation of SQ109)	National Institute of Allergy and Infectious Diseases (NIAID) (United States; April 2009; November 2009; 6 November 2011)
Phase IC study of safety and PK of SQ109 300 mg daily (Randomized; Parallel assignment; Triple (participant, investigator, outcomes assessor); Treatment of TB)	A single dose of SQ109 (300 mg) daily for two weeks; AC: Placebo	**NCT01358162** (Completed: 1: 10: Not available: Safety and tolerability evaluation of SQ109)	NIAID (United States; November 2010; April 2011; 14 May 2013)
Effects of SQ109 on QTc interval in healthy subjects (Randomized; Crossover assignment; None (Open-label); Treatment of TB)	Oral SQ109 (300 mg or 450 mg daily) for seven days; AC: Placebo	**NCT01874314** (Withdrawn due to undisclosed reason; 1; 0; Not available; Effect of SQ109 on QTc interval)	NIAID (United States; Not available; December 2015; 24 March 2014)

## Data Availability

Not applicable.
